# Intermittent theta burst stimulation (iTBS) combined with working memory training to improve cognitive function in schizophrenia: study protocol for a randomized controlled trial

**DOI:** 10.1186/s13063-020-04563-0

**Published:** 2020-07-29

**Authors:** Jiaqi Song, Dan Liu, Meng Zhang, Huiqiu Wang, Shuping Tan

**Affiliations:** 1 Peking University HuiLongGuan Clinical Medical School, Beijing HuiLongGuan Hospital, Beijing, 100096 China; 2 Department of Psychiatry Rehabilitation, Anning Hospital, Shenyang, 110164 Liaoning China

**Keywords:** Cognitive deficits (CD), Intermittent theta burst stimulation (iTBS), Schizophrenia, Working memory training (WMT), Randomized controlled trial (RCT)

## Abstract

**Background:**

Working memory deficit is one of the most critical complex cognitive impairments in schizophrenia. Repetitive transcranial magnetic stimulation (rTMS) is an effective adjuvant therapy, but not still unsatisfactory. Intermittent theta burst stimulation (iTBS), which has recently been used in clinical practice, may have faster and stronger effects comparing the traditional model (10-Hz high-frequency rTMS). A large number of studies have showed that rTMS, especially iTBS, can enhance the neural plasticity of the brain, and cognitive training can improve the cognitive function of schizophrenia. Is there any facilitation effect of iTBS add on cognitive training (such as working memory training, WMT) on cognitive function enhancement in schizophrenia is still unknown.

**Methods/design:**

The proposed study is designed of a double-center, double-blinded, randomized controlled trial that will include 200 schizophrenia patients between 18 and 45 years of age. The patients will be randomized to four groups, i.e., the study group (iTBS+WMT), WMS control group (iTBS+ Simple Response Training (SRT)), iTBS control group (sham iTBS+WMT), and placebo control (sham iTBS+SRT). The patients will receive 3 min 20 s of real or sham stimulation, followed by a short 1–2-min rest and 40 min of WMT training or SRT immediately. Neuropsychological and clinical symptom assessments, with functional and structural MRI, will be performed on baseline, post-treatment, and 3- and 6-month follow-up periods. The primary outcome is cognitive function measured by the MATRICS Consensus Cognitive Battery (MCCB). The secondary outcomes are changes in neuroplasticity, as measured by MRI and other behavioral assessments.

**Discussion:**

The aim of our study is to explore the facilitation effects of iTBS added on WMT in improving cognitive function of schizophrenia. That means, patients with schizophrenia will benefit more in cognitive function improvement from the combination training mode of “preheating (iTBS stimulation changes the neural activity of working memory-related brain regions) and ironning (working memory training).” And the long-term effects of this combined training model will be assessed at a 6-month follow-up period. In case of a significant improvement of working memory with a prolonged effect, the iTBS combined with WMT protocol could be considered as a first-line clinical protocol in schizophrenia treatment. More broadly, the potential for increased universality and efficiency of rTMS with the iTBS model to enhance the neural plasticity of the brain should have a more positive effect on cognitive function in schizophrenia.

**Trial registration:**

chictr.org.cn ChiCTR1900023405. Registered on 25 May 2019

## Background

Schizophrenia is one of the most common severe mental disorders which can seriously affect human health all over the world. The lifetime prevalence rate of schizophrenia is 7.81‰ in China, leading to serious deficiencies or loss of social functions and general life and working skills [1]. This disorder not only causes mental disability, but also brings heavy mental and economic burden of patients, families, and society. Among the causes for disability, cognitive deficit (CD) is one of the most important ones, which has a serious impact on patients’ daily life, work, and disease outcomes [2].

The cognitive impairment of patients with schizophrenia is complicated, among which working memory (WM) deficit is one of the most core and critical cognitive impairments suffered by these patients [3]. Studies have confirmed that central control components play a critical role in working memory system, which are mainly related to dorsolateral prefrontal cortex (DLPFC) function. Meanwhile, the basal ganglia and hippocampus are also involved in working memory processing [4, 5]. The abnormal functional connection between the prefrontal lobe and other working memory-relevant areas may be a vital neurological basis for the deficit of working memory in schizophrenia [6, 7]. MRI shows that the thickness of the left DLPFC cortex and the volume of gray matter decreased in schizophrenic patients [8], and the volume of gray matter decreased in the prefrontal lobe [9] was significantly correlated with the performance of working memory. In addition, diffusion-weighted imaging (DTI) reveals impaired white matter in uncinate fasciculus, the main area of frontotemporal junction in schizophrenic patients, which is significantly correlated with impaired working memory and daily function [10].

Large-scale multi-center trials have shown that cognitive deficits in schizophrenia patients can be improved by repeated intensive and targeted cognitive training, such as cognitive remediation therapy (CRT) [11, 12]. Although a mass of research has confirmed that cognitive remediation therapy and other neuropsychological trainings can significantly improve the cognitive function of schizophrenia patients, the overall effect value is about 0.4, which amounts to a moderate effect level, especially for working memory, which is the core basic cognitive deficit. The improvement effect is lower than that of general cognition, only about 0.35. The recent systematic review of the effects of cognitive training shows that the overall effect of cognitive training is 0.38 and the working memory effect is 0.29 in schizophrenia [13]. Hence, it can be seen that the clinical effect of cognitive training needs to be improved.

Numerous studies have shown that cognitive training plays a role by altering the brain structural and functional plasticity [14]. Brain structural plasticity includes improved cortical thickness [15] or increased white matter integrity [16]. And the functional plasticity includes brain functional changes [17] and cerebral blood flow [18]. So far, repetitive transcranial magnetic stimulation (rTMS) is deemed to be one of the most effective and safe non-drug methods to improve cerebral neural plasticity. It can change the activity of cortical nerve temporarily or continuously and enhance the neural plasticity. However, the recent results are inconsistent. A large sample, multi-center, randomized controlled study recently has found that rTMS at 10 Hz for three consecutive weeks can improve some aspects of cognitive performance, but there is no significant improvement compared with the Sham-stimulation group. It is suggested that the improvement of cognitive function by simple conventional rTMS is limited [19]. At the same time, the clinical conventional rTMS is relatively long-time, generally takes about 20–30 min each session, and the clinical efficiency on cognitive function is not clear, which limits its application on improving cognition of schizophrenia.

However, with regard to recent scientific literature, a newer form of rTMS called intermittent theta burst stimulation (iTBS) has been developed, which simulates burst discharges of physiological action potentials in the central nervous system by cluster stimulation. Compared with conventional rTMS, it can regulate the activity of potential regions in a shorter time and produce stronger and more lasting post-stimulation effects [20]. Theta burst stimulation (TBS), a new stimulation method based on repetitive stimulation, can better simulate the true action potential of human neurons. By regulating synaptic plasticity, TBS produces a rapid and efficient response to the human motor cortex. The major long-term forms of synaptic plasticity, referred to as long-term potentiation (LTP) and long-term depression (LTD), are induced by changes in the postsynaptic Ca2+ concentration [21]. Short bursts of iTBS stimulation, mimicking a common pattern of hippocampal pyramidal neuronal discharge, induce LTP in the hippocampus only when the stimulation is repeated at intervals less than 2 s [22]. And intermittent TBS, which denotes short trains of intermittent bursts, leads to the Ca2+ influx-related excitatory effect (through postsynaptic NMDA receptors) and produces the LTP-like effect [23]. There is less research about iTBS, a new model, compared with conventional rTMS in psychiatric disorder. A retrospective study conducted by Bakker in 2014 found that iTBS (expending 6 min) and conventional rTMS (expending 30 min) had similar effects on the dorsal prefrontal cortex [24]. In the RCT study with higher evidence intensity, Blumberger team has found that iTBS improved depression in patients with treatment-resistant depression and was not inferior to conventional 10-Hz rTMS, which was more efficient in time efficiency (4 min vs 37.5 min). Both treatments have lower shedding rates and similar side effects, with both good safety and tolerability [25].

Although rTMS improves the neuroplasticity of the brain, the improvement of cognitive function is frequently limited to some extent. It is necessary to combine other methods, such as cognitive training, to boost cognitive functions. A research about the combination of rTMS and motor rehabilitation has testified that the intervention based on improving cortical plasticity is more effective than other groups. However, a recent systematic review on the improvement of working memory in schizophrenia suggests that the combination of rTMS and working memory training (WMT) may improve the working memory ability [3].

So far, traditional cognitive training, especially working memory training, has small to medium effect on the improvement of cognitive function. Although standard rTMS can improve the neural plasticity, there are some limitations, such as inefficiency, time-consuming, and lack of strong evidence of cognition improvement. To sum up, we hypothesize that there is a joint enhancement effect of combination between iTBS and WMT, i.e., the combination training mode of “preheating (iTBS stimulation changes the activity of working memory-related brain regions) and ironning (working memory training).” At present, there is no relevant research report to explore whether and how the joint intervention will have a combined effect on improving cognitive function, especially working memory, in schizophrenic patients.

To our knowledge, this is the first study to test the efficacy of a combination of iTBS and WMT on working memory and other cognitive functions of schizophrenia using a double-blinded, randomized controlled trial design. In order to evaluate the efficacy of invention, we will use behavior outcomes (such as neuropsychological assessment) and neuroplasticity outcomes (such as brain microstructural index) combining with neuroimaging indexes. This study will provide scientific evidence for improving cognitive deficits, especially working memory deficits, and elucidate the neuroplasticity mechanism of iTBS stimulation combined with working memory training.

## Methods/design

### Study design

The study will be implemented as a double-center, double-blinded, randomized trial. This was registered under Chictr.org.cn (ChiCTR1900023405). This study will be reported in accordance with both the Consolidated Standards of Reporting Trials (CONSORT) statement and the CONSORT statement for non-pharmaceutical interventions [26, 27].

The primary objective is to explore the facilitation (enhancement) effects of iTBS-induced neuroplasticity changes on the direct and migration effects of working memory training on the cognitive function of schizophrenia. The secondary objectives are to investigate the effects of iTBS, working memory training on brain functional and structural plasticity in schizophrenia, and the facilitation (enhancement) of iTBS on brain functional and structural plasticity induced by working memory training.

### Participants

Two hundred patients with schizophrenia will be recruited on fulfillment of the inclusion criteria. The patients will be randomly allocated into four groups, i.e., the study group (iTBS+WMT), WMS control group (iTBS+ Simple Response Training, SRT), iTBS control group (sham iTBS+WMT), and placebo control (sham iTBS+SRT). Patients will be recruited in two specialty neurostimulation centers at Beijing Huilongguan Hospital, Shenyang Anning Hospital.

This trial has been approved by the local Ethics Committee and followed close to the principles of the Declaration of Helsinki (version 2004). And Standard Protocol Items: Recommendations For Interventional Trials (SPIRIT) Checklist is detailed in additional file according to requirements. All of the patients sign a written consent form in order to take part in the study, after acquainting the aim and procedures.

### Inclusion criteria

- Conformity with the diagnostic criteria of schizophrenia in the fourth edition of the Diagnostic and Statistical Manual of Mental Disorders (DSM-IV) [28]
- Single item score of Positive And Negative Syndrome Scale (PANSS) positive scale was < 5; total score of PANSS positive scale was < 22
- Eighteen to 45 years of age and more than 5 years of formal education
- Voluntarily participate in the study and sign consent form
- The type and dosage of drugs not be adjusted in the past month and in the next month
- Cognitive impairment exists: the length of number backwards ≤ 6
- Right handedness determined by Edinburgh Handedness Assessment Scale

### Exclusion criteria

- Patients with intellectual disability (ID) or neurocognitive disorders
- Severe recession or impulsive excitement, uncooperative
- Severe depression, anxiety, and substance abuse
- Hearing or visual perception disorders
- Severe physical diseases or side effects of medicine, unable to carry out cognitive training
- Pregnant or lactating women

### Dropping criteria

- Total ten sessions not to finish the training among four groups
- The relapse or aggravation of the disease needing the replacement of antipsychotic drugs

### Randomization

Participants will be randomly allocated to four groups: study group (iTBS+WMT), WMS control group (iTBS+SRT), iTBS control group (sham iTBS+WMT), and placebo control (sham iTBS+SRT) in a ratio of 1:1:1:1, with stratification. After patients have given their consent form, randomization will be performed by an independent and professional statistician who is blinded to the patient interventions using block randomization in R Programming (Mathsoft company, USA). Each center has its own enrollment time as a compatibility factor, and 8 patients in the vicinity of the enrollment time are used as a block. There are 15 blocks (total 120 patients) in Beijing Huilongguan Hospital and 10 blocks (total 80 patients) in Shenyang Anning Hospital. Then, the sealed randomization codes and intervention number are sent out to each hospital. Blinding will have to be broken only if a patient needs emergency handling. Once in this way, the participant will be managed as off-trial.

### Blinding

Patients, research psychiatrists, radiologists, statisticians, and neuropsychologists who measure the primary and second outcomes will be blinded to the randomization status from beginning to end. Participants will not distinguish the group they belong to, will not have precise description of iTBS and training parameters, and will not be allowed to speak to each other. Meanwhile, blinding will be maintained for outcome assessment, data management, and analysis.

### Follow-up

Investigators will meet participants on the baseline, ending of trials, and 3 and 6 months after the last treated session. A concerned call is needed to keep in contact with patients and remind them of the next interview in order to avoid being lost to follow-up.

### Intervention

#### The iTBS+WMT group: the study group

**Fig. 1:**
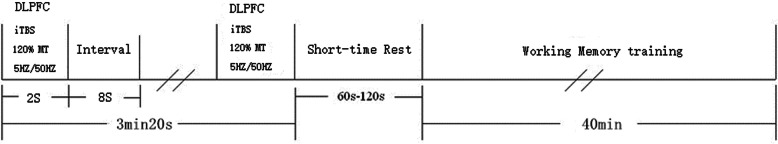
The iTBS+WMT group

During each treatment, the patient received 3 min and 20 s of iTBS stimulation, followed by a 1–2-min short-time rest and 40 min of working memory training immediately (Fig. 1).

iTBS (provided by YiRuiDe CCY-II therapeutic instrument, Wuhan, China) used standard international-approved parameters (120% RMT stimulation intensity or 40% intensity; triplet 50 Hz bursts, repeated at 5 Hz; 2 s on and 8 s off; repeat 20 times; 600 pulses per session; total duration of 3 min 20 s) [25, 29, 30]. After professional testing, the magnetic field intensity has reached the effective range. The left dorsolateral prefrontal cortex is located in each patient with a positioning hat, which was previously proved as optimal target on the basis of clinical finding and whole brain MRI [31]. Initial treatment comprised 30 sessions in total, which consisted of once-daily sessions (on weekdays; i.e., five sessions a week).

The WMT training tool belongs to a computer training system based on the working memory theory model and cognitive processing process that is self-developed and validated by pre-clinical studies. This set of training shows the most typical working memory paradigm, that is, the subjects try to memorize a series of continuous materials (including numbers, languages, pictures) of random length (length is not fixed), and judge the sequence of the last 2–4 materials after presentation. The total number of working memory training sessions is 30, 40 min each, five times a week for 6 weeks.

#### The iTBS+SRT group: the WMT control group

**Fig. 2:**
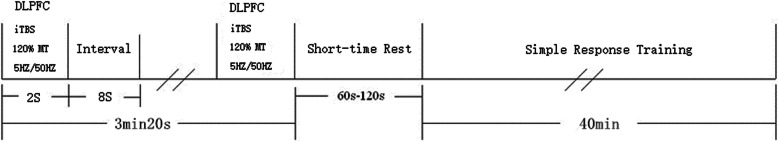
The iTBS+SRT group

The iTBS stimulation method is the same as that of the study group. It is not to practice WMT after the end of the stimulation, but instead to use simple response training (Fig. 2). For example, when a specific shape (such as a red triangle) exists in many sequentially presented geometries, the patient needs to press a key (space). In order to avoid the influence of the material on the result, the material of SRT is exactly the same as that used in WMT training, and this process has no working memory processing content, so it can be used as a comparison condition of WMT.

#### The sham iTBS+WMT group: the iTBS control group

**Fig. 3:**
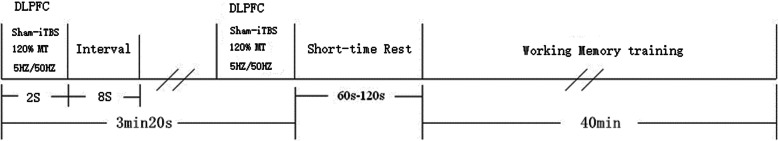
Sham iTBS+WMT group

During each treatment, the patient performs about 3 min and 20 s of sham iTBS. On sham stimulation, a plastic model with the same shape as the coil is fixed on the real coil. This coil has a magnetic shield that reduces the diffusion of the magnetic field under its surface. The distance between the model and the real coil is about 6 cm, which makes the magnetic field generated by the coil basically disappear on the scalp surface. The magnetic field intensity is almost 0 Tesla. So it cannot induce any significant physiological transcranial effect on the brain. After stimulation, the participants perform working memory training at a short interval (1–2 min), and the training method and duration are exactly the same as those of the study group (Fig. 3).

#### The sham iTBS+SRT group: the placebo control

**Fig. 4:**
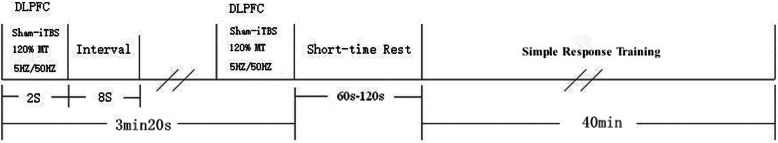
The sham iTBS+SRT group

During each treatment, the iTBS stimulation method is the same as that of the iTBS control group. After stimulation, the participants perform simple response training at a short interval (1–2 min), which is exactly the same as those of the WMT control group (Fig. 4).

Implementing intervention will not require alteration to usual care pathways (minimize changes in dose of medications).

### Primary outcome measures

The primary outcome measures are cognitive function measured by the MATRICS Consensus Cognitive Battery (MCCB).

### Secondary outcome measures

The secondary outcomes are changes in neuroplasticity, as measured by MRI including plasticity and functional plasticity of brain structures and other behavioral assessments. Structural plasticity refers to the analysis of brain gray matter and white matter structure, while functional plasticity includes task-related brain function activation magnitude and task-related functions. Other behavioral assessments include Positive And Negative Syndrome Scale (PANSS), Brief Negative Symptom Scale (BNSS), Personal and Social Performance Scale (PSP), Temporal Experience of Pleasure Scale (TEPS), Self-Esteem Scale (SES), and the visual analogue scale (VAS) of side effects.

### Data collection

At baseline, the following data will be collected: demographic data (age, gender, education, and occupation and so on), smoking, the current medication, concomitant medications, results from a detailed physical examination and neurological examination, and neuropsychological assessment; inclusion and exclusion criteria will be evaluated with electronic case report form (eCRF) by Qing Zhao. The brain functional and structural MRI will be performed, which just conducted in Beijing Huilongguan Hospital. Because there is no MRI equipment, patients in Shenyang Anning Hospital will not do MRI scanning. In order to compare the difference of brain function between patients and normal people, 50 normal subjects matched with patients of age, sex, and education were selected before treatment for the same MRI imaging in Beijing Huilongguan Hospital.

**Fig. 5:**
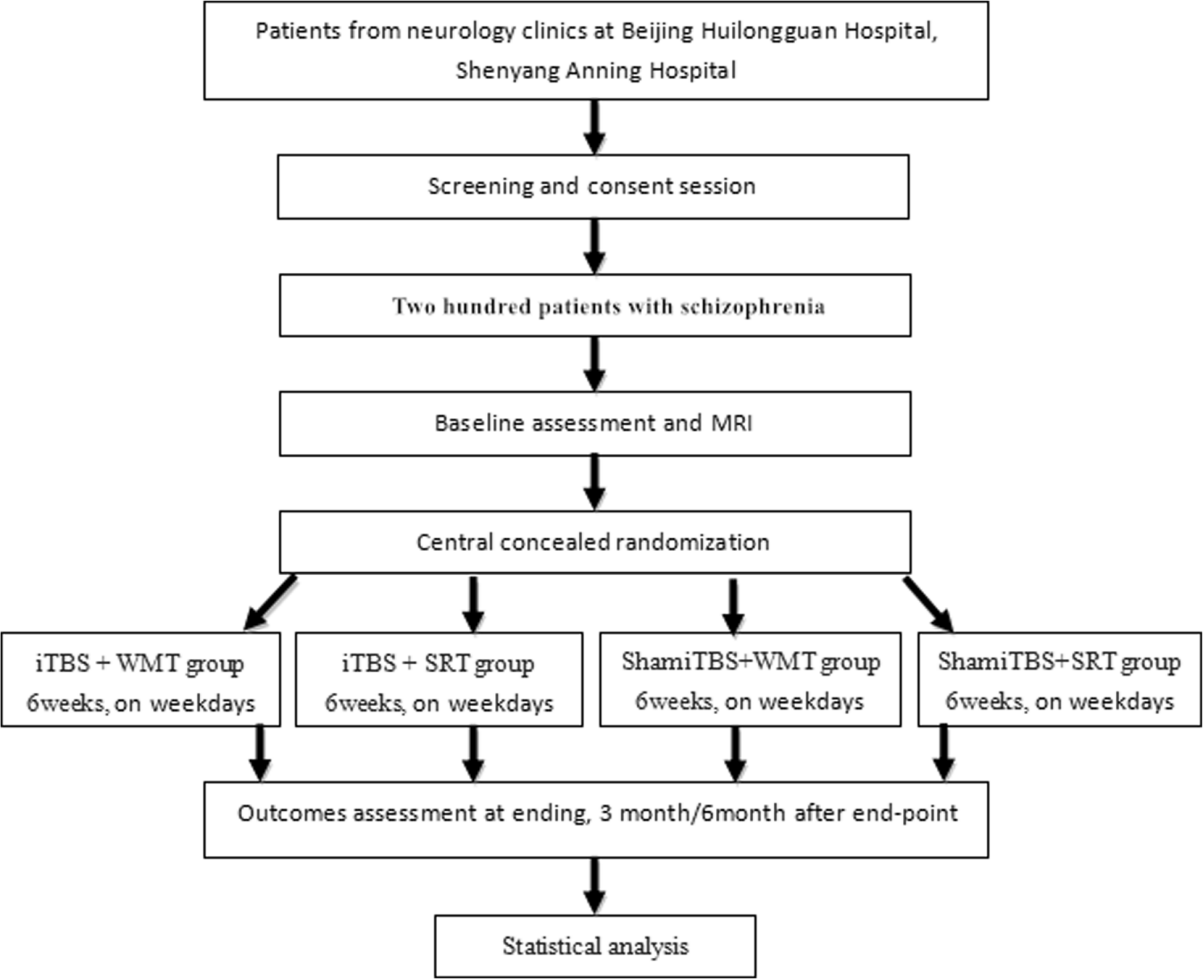
Overview of the flow of participants through the trial

A follow-up assessment will be scheduled at three time points: post-treatment (after 6 weeks of cognitive training) and 3 months and 6 months after end-treatment point. Data from the following examinations will be collected: neuropsychological assessment and functional and structural brain MRI (just for patients in Beijing Huilongguan Hospital) (Fig. 5). During the whole trial period, we will give participants some subsidies to increase their enthusiasm for participating in the study to promote successful completion and follow-up.

### Neuropsychological assessment

1. Cognitive function: MATRICS Consensus Cognitive Battery (MCCB) and Wechsler Adult Intelligence Test Brief Version (WAIS-R) as special measures for evaluating cognitive function in schizophrenia.
  1.1 Evaluation indicators of primary training effect (working memory): spatial span, digital sequence, verbal memory (Hopkins Verbal Learning and Memory Test (HVLT)), visual memory (Brief Visuospatial Memory Test (BVMT)).
  1.2 Evaluation index of migration effect:
    1.2.1 Psychomotor speed: symbol code; trial making A; category fluency
    1.2.2 Reasoning and problem solving: maze test
    1.2.3 Social cognition: emotion management, verbal emotion recognition test (VERT), face emotion recognition test (FERT)
    1.2.4 Attention: continuous performance test (CPT)
2. Clinical symptoms: the Chinese version of the Positive and Negative Symptoms Scale (PANSS), the Concise Negative Symptoms Scale (BNSS), and the Clinical Global Impression (CGI) were used to evaluate clinical symptoms through the semi-fixed inspection method. These were assessed by four clinical training senior physicians in each center.
3. Social function: Personal and Social Performance Scale (PSP)
4. Others: Quality of Life Scale (SQOLS), Temporary Pleasure Experience Scale (TEPS), Self-esteem Scale (SES), the visual analogue scale (VAS) of side effects

All the above scales were evaluated blindly by the raters, who were unaware of the treatment methods used by the patients. Except for the WAIS-R, which was evaluated only once at the baseline, the other scales were assessed at four time points (before and after the training and 3 and 6 months of follow-up).

### MRI protocol

Patients will finish brain MRI at baseline and 6 weeks after whole training. MRI will be performed using an optimized protocol, using the 3.0-T MRI scanner (Siemens Healthcare, Prisma, Germany). Functional MRI data collected will be analyzed using the Statistical Parametric Mapping (SPM) (UCL Queen Square Institute of Neurology, London, UK). Brain activation and connectivity changes between the baseline and ending of training will be compared among four groups.

### Outcome assessment

All participants from two hospitals will be assessed by neuropsychologists who have received consistent training, and brain MRI examinations will be performed using the same MRI scanner.

### Data management

This experiment will keep the data strictly confidential and stored in Alibaba Cloud through eCRF, and each person in our team has a personal account for data entry and revises. The background of eCRF has a correction function to prevent some errors. This trial collects behavioral data and nuclear magnetic data, not biological data. The datasets analyzed and the informed consent form during the current study are available from the corresponding author on reasonable request.

### Data monitoring

The project management group is composed of Jiaqi Song and Huiqiu Wang. The group is mainly responsible for managing the overall work of the two centers and reviewing every 6 weeks. The trial auditing group is composed of two staff members of the audit department and is responsible for regularly reviewing the progress, authenticity, and safety every 6 weeks. Dan Liu is independently responsible for all data monitoring of the study. The intervention may produce mild adverse events, such as headache and irritability, which can be improved by giving the patient rest or interval stimulation. If the patients show unbearable, they can opt out of the trial. We should report this situation to the supervisory department for record. The trial auditing group and the independent Data Monitoring and Ethics Committee have to review conduct throughout the trial period.

### Sample size

The hypothesis is 77.2% response rate in the cognitive training group and 41.4% in the control group on our preliminary studies [32]. The difference between the two groups was improved about 35.8%, as the basis for calculation. With a statistical power of 80% and a significance level of 5%, the minimum sample size of 28 subjects is required in each group. With our previous research experience, we allow for a maximum dropout rate of 20%. The study adopts the block randomization to divide into groups with 8 patients in each block. The actual minimum sample size of each group was 34 cases. Combining each center clinical states, Beijing center inclusion number has been set to 120 patients to participate into this research and Shenyang center has 80 patients.

### Statistical analysis

The analysis will be conducted on the basis of the intent-to-treat (ITT) principle. Some descriptive analysis of the data gathered during each assessment will be carried out precisely until the final assessment. Special time points for analysis are the baseline, end of iTBS or sham iTBS sessions, and 3 and 6 months after the final treatment. Median and range describe continuous variables, and frequencies and percentages describe qualitative variables.

We will accept a significance level of 5% (*P* < 0.05) corrected for multiple comparisons when needed. To assess our primary endpoint criterion, i.e., cognitive performance changes on the task, outcomes at four time points will be compared using repeated measurement analysis of variance of variance with time (baseline, ending, and 3 and 6 months after the final treatment) and group (study group (iTBS+WMT), WMS control group (iTBS+SRT), iTBS control group (sham iTBS+WMT), and placebo control (sham iTBS+SRT)) as factors. Further comparison between the two groups can show the simple iTBS effect, the simple WMT effect, and the facilitation effect of iTBS on WMT. Sensitivity analyses will explore the impact of imputation of data losses including loss to follow-up and dropout.

In the case that the data do not meet the normal distribution assumption, the non-parametric Mann-Whitney test will be used to compare performance improvements between four groups. SPM will be used to analyze imaging data to detect any changes in brain function and structure caused by cognitive training.

## Discussion

To our knowledge, this is the first trial aiming to solve the problem of limited overall effect and migration effect of working memory training in schizophrenia by performing professional training, which is based on changing the neuroplasticity of working memory-related brain areas through iTBS primitively.

This trial has several advantages. First, this study use iTBS stimulation, which is currently a well-evidentiary and efficient way to alter the plasticity of the brain, as a tool to regulate the functional state of brain regions before working memory training. The effects of iTBS on brain function and structural plasticity in schizophrenia were investigated by structural and functional MRI.

Second, we hypothesize that iBTS stimulation, which will be able to alter brain plasticity, will facilitate (enhance) the clinical effects of working memory training on improving cognitive function of schizophrenia? This is a key issue in the study of WMT mechanism, that is, whether the way of “preheating (iTBS stimulation activates local neuronal activity first) and then ironning (working memory training)” can make “iron better tempered” (the changes of brain function and structure are more significant). In our study, the WMT control group (iTBS+SRT) and iTBS control group (sham iTBS+WMT), were set up to compare with the study group, respectively, and to analyze the differences of brain plasticity, to explore whether the training of “preheating and then ironning” (iTBS+WMT) is superior to that of “ironning without preheating” (sham iTBS+WMT) and “ironning without preheating” (iTBS+SRT) in changing the neuroplasticity (function and structure) and to provide a scientific basis for exploring ways to enhance the neuroplasticity of the brain and improve its function.

Another advantage of this trial is to explore the migration effect, that is, whether training can improve untrained cognitive ability as well as related-cognitive processing ability, such as whether it can improve logical reasoning ability after working memory training? The training for the normal youngs suggests that working memory training alone can improve memory function and fluid intelligence, thus verifying the existence of migration effect of working memory training. However, there are few studies on whether the migration effect occurs in schizophrenia, and this study makes up for the shortcomings in this area.

This sample cannot entirely represent a population combining comorbidities with considerable treatment heterogeneity. However, we opted to the rigorous inclusion, exclusion, and dropping criteria in order to acquire fewer confusing variables and make trial more accuracy and truthfulness.

## Trial status

Patient recruitment is currently ongoing. This is the first version of this protocol (V1.0). If the protocol needs to be amended, we should notify firstly the sponsor and the centers. After passing the ethics committee review, a copy of the revised protocol will be sent to the managers of two centers and be added to the web for record accurately. The first patient has been enrolled on December 23, 2019, and it is expected to finish by the end of 2020. Due to the impact of COVID-19, our related researches have been severely affected, and the end time will be appropriately postponed.

## Supplementary information

**Additional file 1.** SPIRIT 2013 Checklist: Recommended items to address in a clinical trial protocol and related documents.

**Additional file 2.** Funding Documentation: National Natural Science Foundation of China General Projects.

**Additional file 3.** Ethical review approval

## Data Availability

The datasets are available from the corresponding author on reasonable request.

## Abbreviations

rTMS: Repetitive transcranial magnetic stimulation
iTBS: Intermittent theta burst stimulation
CONSORT: Consolidated Standards of Reporting Trials
MRI: Magnetic resonance imaging
LTP: Long-term potentiation
LTD: Long-term depression
WMT: Working memory training
SRT: Simple Response Training
MCCB: MATRICS Consensus Cognitive Battery
PANSS: Positive And Negative Syndrome Scale
WAIS-R: Wechsler Adult Intelligence Test Brief Version
BNSS: Brief Negative Symptom Scale
CGI: Clinical Global Impression
PSP: Personal and Social Performance Scale
TEPS: Temporal Experience of Pleasure Scale
SQOLS: Quality of Life Scale
SES: Self-Esteem Scale; the visual analogue scale (VAS) of side effects
HVLT: Hopkins Verbal Learning and Memory Test
BVMT: Brief Visuospatial Memory Test
VERT: Verbal Emotion Recognition Test
CPT: Continuous Performance Test
SPIRIT: Standard Protocol Items: Recommendations For Interventional Trials
eCRF: Electronic case report form
ID: Intellectual disability
