# Supplementary material for: Intermittent theta burst stimulation (iTBS) combined with working memory training to improve cognitive function in schizophrenia: study protocol for a randomized controlled trial
Source: Trials. 2020 Jul 29;21:683. doi: 10.1186/s13063-020-04563-0 (PMC7387875; doi:10.1186/s13063-020-04563-0)
Supplement: Supplementary file 2 — Additional file 2. Funding Documentation: National Natural Science Foundation of China General Projects. [file 13063_2020_4563_MOESM2_ESM.zip › attached file 1 (Funding Documentation-original edition).pdf]

关于国家自然科学基金资助项目批准及有关事项的通知

谭淑平　　先生/女士:

根据《国家自然科学基金条例》的规定和专家评审意见，国家自然科学基金委员会（以下简称自然科学基金委）决定批准资助您的申请项目。项目批准号: 31671145，项目名称: 脑可塑性对精神分裂症工作记忆训练效应的影响 ，直接费用: 62.00万元，项目起止年月: 2017年01月至 2020年 12月，有关项目的评审意见及修改意见附后。

请尽早登录科学基金网络信息系统（https://isisn.nsfc.gov.cn），获取《国家自然科学基金资助项目计划书》（以下简称计划书）并按要求填写。对于有修改意见的项目，请按修改意见及时调整计划书相关内容; 如对修改意见有异议，须在计划书电子版报送截止日期前提出。**注意: 请严格按照《国家自然科学基金资助项目资金管理办法》填写计划书的资金预算表，其中，劳务费、专家咨询费科目所列金额与申请书相比不得调增。**

计划书电子版通过科学基金网络信息系统（https://isisn.nsfc.gov.cn）上传，由依托单位审核后提交至自然科学基金委进行审核。审核未通过者，返回修改后再行提交; 审核通过者，打印为计划书纸质版（一式两份，双面打印），由依托单位审核并加盖单位公章后报送至自然科学基金委项目材料接收工作组。计划书电子版和纸质版内容应当保证一致。

向自然科学基金委提交和报送计划书截止时间节点如下:

1、提交计划书电子版截止时间为**2016年9月11日16点**（视为计划书正式提交时间）;

2、提交计划书电子修改版截止时间为**2016年9月18日16点**;

3、报送计划书纸质版截止时间为**2016年9月26日16点**。

请按照以上规定及时提交计划书电子版，并报送计划书纸质版，未说明理由且逾期不报计划书者，视为自动放弃接受资助。

附件: 项目评审意见及修改意见

国家自然科学基金委员会
生命科学部
2016年8月17日

附件：项目评审意见及修改意见表

项目批准号	31671145	项目负责人	谭淑平	申请代码1	C2103
项目名称	脑可塑性对精神分裂症工作记忆训练效应的影响				
资助类别	面上项目		亚类说明		
附注说明	常规面上项目				
依托单位	北京回龙观医院				
直接费用	62.00 万元		起止年月	2017年01月 至 2020年12月	

通讯评审意见:
<1>该研究针对精神分裂症患者的工作记忆特征开展相关研究，旨在阐述采用经颅磁刺激（rTMS）结合工作记忆训练，改变脑功能及可塑性，探讨rTMS所致脑可塑性改变对工作记忆训练效应的易化效应，为精神分裂症认知缺陷干预技术提供线索依据。该研究设计拟入组129例精神分裂症患者，随机分入rTMS干预组、rTMS+工作记忆训练组，伪rTMS+工作记忆训练组，并于干预前后给予认知及MRI检测。该研究团队前期有较好的工作基础，病例充足，样本量估算合理，有望在规定期限内完成相应研究和分析。
同意资助。
<2>1.精神分裂症患者的认知损害尤其是工作记忆方面的损害是临床上的一个重要而棘手的问题，本项目旨在探索先使用rTMS改变脑功能及可塑性,再予以工作记忆训练，以期提高训练效应，促进相应脑区功能及结构的可塑性变化，具有较大的应用价值和科学意义。
2.本项目的科学问题比较明确,具有一定的创新性。rTMS和工作记忆训练（WMT）单独运用在改善精神分裂症工作记忆缺陷上的临床应用和相关机制研究虽较多，也有部分涉及二者联用对于认知功能改善，但从神经可塑性角度对二者联用机制的fMRI研究较少。
3.本研究的研究内容较为清晰，可行性好，但在研究方案中存在部分问题，建议改进。工作记忆训练的材料中涉及到言语、数字、图形、空间位置、语音序列，在研究方案中入组标准：“18-45岁，接受5年以上正规教育”，“存在认知损害：数字倒背广度=<6”可能存在以下问题：若被试受教育程度太低，能否正确理解训练的要求？若不对教育程度进行控制，是否会成为混淆变量？
4.申请人个人既往工作主要集中于精神疾病认知功能基础与临床研究工作，研究经历和水平良好。其所在团队在该方面有较好的工作基础，有一定的前期工作发现，团队人员配置合理，技术平台良好，具有可行性。
<3>认知缺陷症状是精神分裂症的核心症状之一，是精神分裂症功能预后的重要影响因素，而认知缺陷症状又是治疗难点。目前国内外围绕精神分裂症认知缺陷症状的治疗展开大量研究，其中认知矫正治疗及rTMS是重要的研究方向。本研究将两者有机结合起来，尤其探讨两者的叠加效应，不仅探讨了其临床效应，而且深入探讨了其神经基础及机制，学术思想具有创新性，项目具有较好的科学意义，研究结果可以实现转化医学目地，用于指导临床，从而形成具有操作可行的治疗方案。
研究内容设计合理，关键问题描述准确，尤其是精心设计了两个对照组，这样既可以分别探讨rTMS与认知训练的单独效应，又可以凸显出两者的叠加效应或整合效应。总体研究方案合理可行，不仅关注了训练本身的效应，而且关注了迁移效应及其机制，在神经机制方面，同时关注了静态、动态，脑灰质及白质，能够比较全面的了解其变化。项目组前期从事了大量研究工作，尤其认知训练方面形成了较好的研究方案和基础，且具有较好的支持系统，具有项目实施的基础，完成项目的条件和能力。
同意优先资助。
<4>本项目拟以精神分裂症患者为研究对象，采用功能性核磁共振成像技术来考察干预前后（rTMS+工作记忆训练）工作记忆及脑结构与功能的变化，从而探讨rTMS引起的脑可塑性改变对工作记忆训练效应的促进作用，为精神分裂症的认知缺陷干预提供科学依据。
申请人长期以来一直从事临床干预技术对精神分裂症认知及症状改变的研究，并且积累了一定的研究成果，为本项目的顺利开展提供了方法上的参考。本项目的顺利开展将（1）有利于阐明对rTMS结合工作记忆训练如何促进精神分裂症认知功能改善的脑机制的理解，并且（2）为精神分裂症的有效临床干预提供方法学方面的参考，具有一定的科学意义和临床价值。但是，本项目尚存在以下待完善之处:
（1）第27-28页“（3）功能可塑性分析”，重点分析之一是静息态下的任务正相关网络和任

务负相关网络，但在立项依据部分并没有提及这两个网络的相关文献。另外，建议增加分析静息态局部功能方面发生的变化（如ALFF，ReHo指标）？
（2）第29页，仅提到结构像和功能像的处理，DTI数据的处理如何进行？请简要说明。
综上，建议可资助。
<5>本项目主要综合采用rTMS、认知训练方法和功能磁共振成像技术，研究精神分裂症患者在工作记忆时脑功能网络的改变，进一步为精分患者的优化的干预训练方案提供脑科学依据。特别是项目在研究rTMS和工作记忆训练的组合干预模式，与其他单一的干预模式比较有效性方面，具有重要创新和临床价值。项目立项依据论述充分，研究方案和技术合理，项目研究团队在精分的临床和神经机制研究方面具有较好的基础，并发表了多篇SCI研究论文，项目研究团队也配置合理。特别是，项目组拟采用multi-band fmri进行扫描，非常值得肯定，这保证了图像数据具有较高的质量。综上，建议优先资助。

对研究方案的修改意见:

生命科学部

2016年8月17日
