# Supplementary material for: Intermittent theta burst stimulation (iTBS) combined with working memory training to improve cognitive function in schizophrenia: study protocol for a randomized controlled trial
Source: Trials. 2020 Jul 29;21:683. doi: 10.1186/s13063-020-04563-0 (PMC7387875; doi:10.1186/s13063-020-04563-0)
Supplement: Supplementary file 3 — Additional file 3. Ethical review approval [file 13063_2020_4563_MOESM3_ESM.zip › attached file 2 (Ethical Approval Document-original edition).pdf]

伦理审查批件

批件号	2018-36-科-修 1		
项目名称	脑可塑性对精神分裂症工作记忆训练效应的影响		
项目来源	国家自然科学基金　编号：31671145		
研究单位	北京回龙观医院		
主要研究者	谭淑平		
审查类别	科研基金项目	审查方式	快速审查
审查日期	2019.12.18		
审查委员	辛衍涛　王绍礼　王志仁		
审查批准文件	审查文件：修正案审查申请报告；研究方案（编号：2.0；版本日期：2019-12-05）；知情同意书（健康人版和患者版）（编号：2.0；版本日期：2019-12-05）<br>批准文件：修正案审查申请报告；研究方案（编号：2.0；版本日期：2019-12-05）；知情同意书（健康人版和患者版）（编号：2.0；版本日期：2019-12-05）		

会议审查意见：同意。

审查批准文件：

根据卫生部《涉及人的生物医学研究伦理审查办法》(2016)、SFDA《药品临床试验质量管理规范（2003)》、CFDA《药物临床试验伦理审查工作指导原则》(2010)、《医疗器械临床试验质量管理规范》（2016）、WMA《赫尔辛基宣言》(2013）和 CIOMS《人体生物医学研究国际道德指南》(2002）和 ICH-GCP 的伦理原则。

经本伦理委员会审查，该项目方案和知情同意书，符合伦理要求。同意开展研究。

北京回龙观医院伦理委员会	盖章
主任委员签字	
日期	2019.12.18
联系电话	010-83024461

北京回龙观医院伦理委员会
