# Supplementary material for: Intermittent theta burst stimulation (iTBS) combined with working memory training to improve cognitive function in schizophrenia: study protocol for a randomized controlled trial
Source: Trials. 2020 Jul 29;21:683. doi: 10.1186/s13063-020-04563-0 (PMC7387875; doi:10.1186/s13063-020-04563-0)
Supplement: Supplementary file 2 — Additional file 2. Funding Documentation: National Natural Science Foundation of China General Projects. [file 13063_2020_4563_MOESM2_ESM.zip › attached file 1 (Funding Documentation-English edition).doc]

**National Natural Science Foundation of China General Projects**

| Project Title | The effect of brain plasticity on the working memory training effect of schizophrenia | | | | | | |
| --- | --- | --- | --- | --- | --- | --- | --- |
| Grant No. | 31671145 | | | | Amount | | RMB 620,000 |
| Disciplinary code | | | C2103 | | | | |
| Start date | January 2017 | | | | Expires | | December 2020 |
| Experts comments:  The research content is reasonably designed and the key issues are described accurately, especially the two control groups are carefully designed. Overall the case is reasonable and feasible. The study not only focuses on the effect of training itself, but also on the migration effect and its mechanism. In terms of neural mechanism, at the same time, it pays attention to static and dynamic process, gray matter and white matter, and is able to understand its changes more comprehensively. The project team engaged in a large number of research work, especially in cognitive training. They have formed a better research program and foundation, and have a better support system, with the basis of the project implementation, in order to complete the project.  Therefore, priority funding was agreed.  National Natural Science Foundation of China  Life Science Department  August 17, 2016 | | | | | | | |
| Principal Investigator | | | | | | | |
| Name in Chinese | | 谭淑平/Shuping Tan | | | | Sex ☑Male □Female | |
| 汉语拼音 | Surname： Tan First name：Shuping | | | | | | |
| Title | Professor | | | Position | | Director of Mental Research Center | |
| Organization | | Beijing HuiLongGuan Hospital, Beijing 100096, China | | | | | |
| Address: Beijing HuiLongGuan Hospital, Beijing 100096, China | | | | | | | |
| Tel. | +86 010-83024485 | | | E—mail | | shupingtan@126.com | |
