# Supplementary material for: Intermittent theta burst stimulation (iTBS) combined with working memory training to improve cognitive function in schizophrenia: study protocol for a randomized controlled trial
Source: Trials. 2020 Jul 29;21:683. doi: 10.1186/s13063-020-04563-0 (PMC7387875; doi:10.1186/s13063-020-04563-0)
Supplement: Supplementary file 3 — Additional file 3. Ethical review approval [file 13063_2020_4563_MOESM3_ESM.zip › attached file 2 (Ethical Approval Document-English edition).doc]

| Approval number | 2018-36-revision 1 | | |
| --- | --- | --- | --- |
| Project title | The effect of brain plasticity on the working memory training effect of schizophrenia | | |
| Project source | National Natural Science Foundation of China | | Number：31671145 |
| Research institute | Beijing Huilongguan Hospital, Beijing, China | | |
| Principal investigator | Shuping Tan | | |
| Review category | scientific research fund projects | Review method | quick review |
| December 18, 2019 | December 18, 2019 | | |
| Review committee | Yantao Xin, Shaoli Wang, Zhiren Wang | | |
| Review and approve documents | Review documents: research program (Number 2.0, version date: 2019-12-05), Informed consent form (healthy controls and patients) (Number 2.0, version date: 2019-12-05).  Approve documents: research program (Number 2.0, version date: 2019-12-05), Informed consent form (healthy controls and patients) (Number 2.0, version date: 2019-12-05). | | |
| Review opinions | agreement | | |
| Review and approve documents:  According to the ethical principles of Ethical review methods for human biomedical research (2016), Criterions for the Quality Control of Clinical Trial of drugs (2003), Guiding principles for ethical review of drug clinical trials (2010), Medical Device Clinical Trial Quality Management Specification (2016), Declaration of Helsinki (2013), International Moral Guide to Human Biomedical Research (2002), Guidelines for the management of clinical trials.  After review by the ethics committee, the project program and informed consent met the ethical requirements. The committee agrees to carry out research. | | | |
| Ethics Committee of Beijing Huilongguan Hospital | unit seal | | |
| The chairman of the ethics committee | Yantao Xin | | |
| December 18, 2019 | December 18, 2019 | | |
| The phone number | 00-86-010-83024461 | | |

**Ethical Review** **Approval**
